# Sparse connectivity enables efficient information processing in cortex-like artificial neural networks

**DOI:** 10.3389/fncir.2025.1528309

**Published:** 2025-03-13

**Authors:** Rieke Fruengel, Marcel Oberlaender

**Affiliations:** ^1^In Silico Brain Sciences Group, Max Planck Institute for Neurobiology of Behavior-caesar, Bonn, Germany; ^2^International Max Planck Research School (IMPRS) for Brain and Behavior, Bonn, Germany; ^3^Center for Neurogenomics and Cognitive Research, Department of Integrative Neurophysiology, VU Amsterdam, Amsterdam, Netherlands

**Keywords:** connectivity, structure–function, cortex, artificial neural networks, recurrent, sparse

## Abstract

Neurons in cortical networks are very sparsely connected; even neurons whose axons and dendrites overlap are highly unlikely to form a synaptic connection. What is the relevance of such sparse connectivity for a network’s function? Surprisingly, it has been shown that sparse connectivity impairs information processing in artificial neural networks (ANNs). Does this imply that sparse connectivity also impairs information processing in biological neural networks? Although ANNs were originally inspired by the brain, conventional ANNs differ substantially in their structural network architecture from cortical networks. To disentangle the relevance of these structural properties for information processing in networks, we systematically constructed ANNs constrained by interpretable features of cortical networks. We find that in large and recurrently connected networks, as are found in the cortex, sparse connectivity facilitates time- and data-efficient information processing. We explore the origins of these surprising findings and show that conventional dense ANNs distribute information across only a very small fraction of nodes, whereas sparse ANNs distribute information across more nodes. We show that sparsity is most critical in networks with fixed excitatory and inhibitory nodes, mirroring neuronal cell types in cortex. This constraint causes a large learning delay in densely connected networks which is eliminated by sparse connectivity. Taken together, our findings show that sparse connectivity enables efficient information processing given key constraints from cortical networks, setting the stage for further investigation into higher-order features of cortical connectivity.

## Introduction

Cortical networks are very sparsely connected. In fact, we recently showed that in a given subvolume of sensory cortex, <1% of neurons with overlapping axons and dendrites will form a synaptic connection (Udvary et al., 2022). What is the relevance of such sparse connectivity for a network’s function? Surprisingly, sparse connectivity was shown to impair information processing in artificial neural networks (ANNs), making training more difficult and leading to worse performance (Evci et al., 2019). Do these findings in ANNs imply that sparse connectivity also impairs information processing in biological neural networks, or are there conditions under which sparsity may be beneficial?

Although the ANNs underlying much of modern deep learning were originally inspired by the brain, there are major differences between conventional ANNs and biological neural networks. While neurons are arranged in layers consisting of different cell types, there are abundant recurrent connections within and between layers [reviewed in Singer (2021)]. Meanwhile, conventional ANNs are typically initialized with dense, feedforward connectivity, meaning that each node in a given layer is connected unidirectionally to all nodes in the subsequent layer. In the brain, a neuron is either excitatory or inhibitory (Dale’s law; Eccles, 1976), whereas conventional ANNs place no such constraints on weights, allowing individual nodes to have both positive and negative outgoing connections. Determining the relevance of these differences in structure for network function is challenging. It may seem an obvious approach to take the empirically measured connectivity from, e.g., a dense electron microscopic reconstruction, and use it to construct an ANN replica with biologically realistic connectivity. Unfortunately, such a detailed replica of cortical connectivity is challenging to compare to other architectures in order to understand which structural properties are actually relevant for function. How can we isolate the effect of individual features of cortical networks on information processing in a network?

To this end, we systematically generate and train artificial neural networks (ANNs) constrained by selected, interpretable features of cortical network architecture. We constructed ANNs with different degrees of sparsity in the hidden layers – the sparser the network, the fewer nodes are connected to each other by a trainable weight. Connectivity in cortex is highly recurrent, so we compared the effect of sparsity in feedforward ANNs and recurrent neural networks (RNNs). It has been suggested that the degree of sparsity in cortex is affected by the size of the brain, with larger brains having sparser connectivity (Herculano-Houzel et al., 2010), so we here investigated sparsity and recurrence in networks of different sizes. Our networks were trained by backpropagation and gradient descent. We find that in networks which are large and recurrent, like cortical networks, sparse connectivity enables networks to achieve better performance when training time or data are limited than conventional, dense connectivity.

An important function of sensory areas in the cortex is to encode inputs in a way that enables them to be distinguished by downstream areas. Similarly, ANNs must encode their inputs in the activations of nodes in the hidden layers in order to allow classification at the output layer. We therefore next investigated how our ANNs represent their inputs in the activations of the hidden layer nodes, and to what extent connectivity in the hidden layer affects this input representation. We find that although in the case of large, recurrent networks, both sparsely and densely connected networks form sparse representations of their inputs, sparse networks use more distributed representations which are more robust to neuronal noise.

Finally, we constructed and trained ANNs which obey Dale’s principle, which states that a neuron releases the same set of neurotransmitters at all of its synapses (Eccles, 1976); broadly, in cortical networks, this means that each neuron is either excitatory or inhibitory and must remain that way. For ANNs, this corresponds to each node having exclusively positive or negative outgoing weights, and this sign remaining unchanged throughout the training process. It has been found that applying such constraints to conventional ANNs often impairs their training (Cornford et al., 2020). Here, we constructed Dale-compliant RNNs with a proportion of inhibitory nodes corresponding to the proportion of inhibitory neurons reported in sensory cortex (Meyer et al., 2011). We find that the training of densely connected networks is indeed severely slowed by Dale’s principle, but that sparse connectivity enables Dale’s networks to train almost as efficiently as their unconstrained counterparts.

## Results

### Sparse connectivity enables efficient training of large and recurrent networks

**Figure 1:**
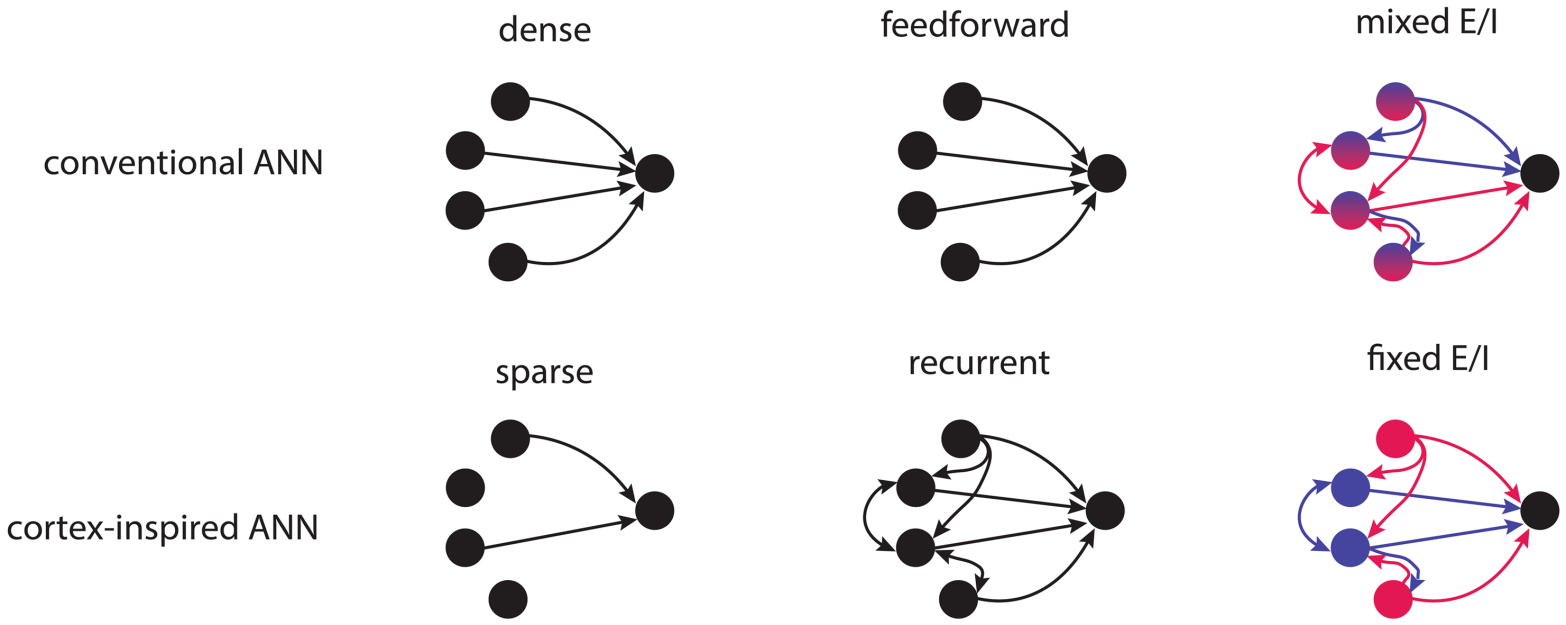
Schematic illustration depicting ANNs with conventional or cortex-inspired structural properties. Top row, left to right: conventional ANNs are densely connected, feedforward and have nodes with mixed excitatory and inhibitory weights. Bottom row, left to right: cortex-inspired ANNs are sparsely connected, have recurrent connectivity and have nodes with fixed excitatory or inhibitory weights.

**Figure 2:**
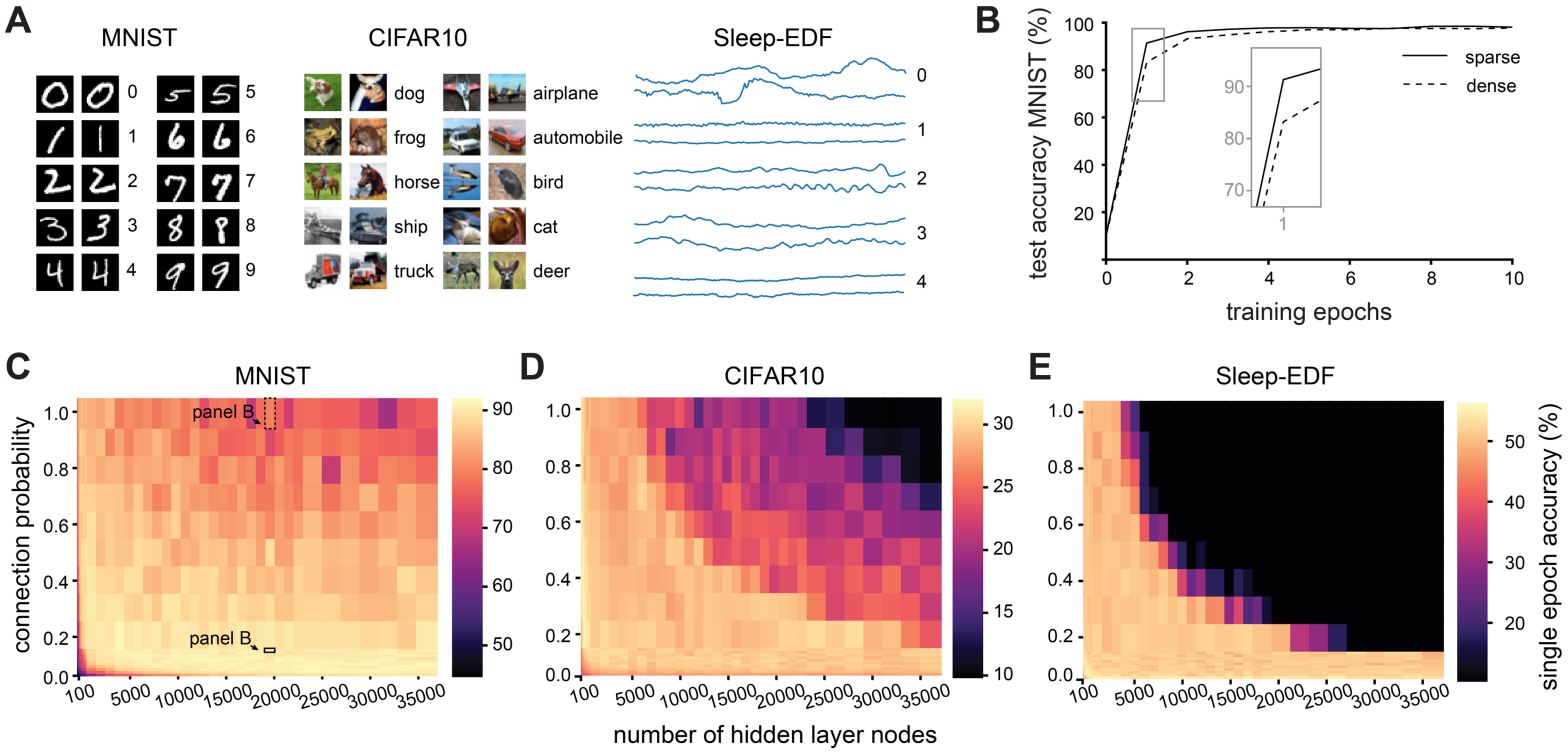
Effect of network size and sparsity on time-limited training. **(A)**. Examples from each of the three classification datasets used in this study. **(B)**. Test accuracy over 10 training epochs of a densely (connection probability = 1) and sparsely (connection probability = 0.1) connected network with 20,000 hidden layer nodes on the MNIST dataset. **(C)**. Test accuracy after one training epoch on the MNIST dataset for networks with different numbers of hidden layer nodes and connection probabilities between nodes in the hidden layer. **(D)**. Test accuracy after one training epoch on the CIFAR10 dataset for networks with different numbers of hidden layer nodes and connection probabilities between nodes in the hidden layer. **(E)**. Test accuracy after one training epoch on the Sleep-EDF dataset for networks with different numbers of hidden layer nodes and connection probabilities between nodes in the hidden layer.

We generated networks with different connectivity parameters to systematically investigate the effect of network size, sparsity and recurrence on training (Figure 1). We began by training RNNs with different numbers of hidden layer nodes and different connection probabilities between these nodes on MNIST handwritten digit recognition (Lecun et al., 1998), a popular benchmark machine learning task (Figure 2A). Each of our networks has a single hidden layer containing all of the recurrently connected nodes, which may be sparsely connected to each other, as well as a fully connected input and output layer (Supplementary Figure S1). The lower the connection probability, the fewer nodes were connected by a trainable weight, and therefore the sparser the network. As the connection probability is applied to connectivity between all nodes in the hidden layer, the connection probability is functionally equivalent to the network density in our networks (see also Methods). Since RNNs, like biological neural networks, process information with a temporal dimension, we modified the image-based MNIST dataset to be compatible with recurrent networks by encoding each image as a time series (Supplementary Figure S1). To avoid interpreting artefacts from a particular initialization, we repeated each set of network connectivity parameters 10 times, using different random weight initialisations.

Our first observation was that networks with different connectivity parameters differ in their performance most notably during the early stages of training (Figure 2B). To compare the performance of networks with limited training time, we therefore evaluated the single epoch accuracy, i.e., the performance on the testing dataset after the network has seen each example in the training dataset only once. For the purposes of this study, we define as “sparse” any network with 10% connectivity or less. We find that sparse connectivity facilitates time-limited learning in large and recurrently connected networks, but confers no benefit and is rather detrimental in small networks (Figure 2C). After just a single epoch of training, large sparse networks (with 20,000 nodes and a connection probability of 10%) attained a mean test accuracy of of 90.3 ± 2.0% while their densely connected counterparts only reached 77.5 ± 7.3% (for all values see Supplementary Table S1). This disparity in performance is abolished by further training, with sparse and dense networks reaching comparable test accuracies after around 80 training epochs (*p* > 0.05, KS-test). Could this finding simply be caused by the different number of parameters between networks with different connectivity levels? To investigate this, while keeping the number of weights in the hidden layer constant, we systematically varied the connection probability of the networks. Hereby, we can compare between larger, sparse networks and smaller, dense networks with the same number of hidden layer weights. We find that even when the number of weights is the same, sparse connectivity allows for better performance in a short training time than dense connectivity (Supplementary Figure S2).

Next, we assessed whether these findings generalize to another, more challenging benchmark image recognition dataset, CIFAR10, which uses full-color RGB images (Krizhevsky, 2012). Similarly to results on MNIST, large sparse networks outperform their dense counterparts on the CIFAR10 dataset (Figure 2D). After a single training epoch, large sparse networks attained a mean test accuracy of 29.7 ± 1.5%, while large densely connected networks only reached 17.4 ± 3.9% (for all values see Supplementary Table S2). On the CIFAR10 dataset, while large sparse networks initially outperform their dense counterparts and attain a higher maximum accuracy, they begin to overfit after around 40 epochs, and their test performance eventually drops below that of dense networks.

To confirm that these findings are not due to some artefact related to performing image classification with recurrent networks, we evaluated the networks on a native timeseries dataset, Sleep-EDF sleep-stage classification from EEG recordings (Kemp et al., 2000). Once again, we find that large sparse networks outperform their dense counterparts, reaching 50.7 ± 1.8% accuracy after one training epoch while dense networks of the same size only attained 10.3 ± 0.0% accuracy (Figure 2E, for all values see Supplementary Table S3). We therefore conclude that sparse connectivity facilitates training when training time is limited in large and recurrent networks, and that this effect is not dataset-specific.

**Figure 3:**
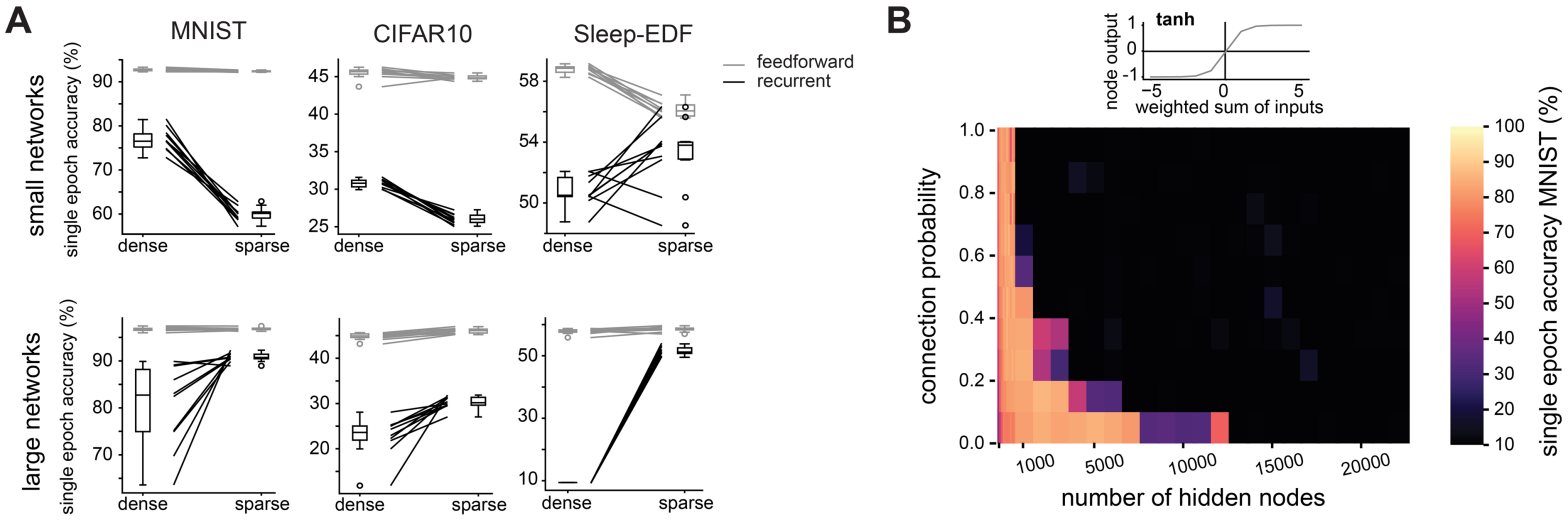
The benefit of sparse connectivity is dependent on a recurrent network architecture and a single-cell activation function with a threshold. **(A)**. Comparison of test accuracy between densely (connection probability = 1) and sparsely (connection probability = 0.1) connected networks in small networks (1,000 hidden layer nodes, top row) and large networks (10,000 hidden layer nodes, bottom row). Nodes were either placed in a single hidden layer with recurrent connectivity (black lines) or in two hidden layers connected by feedforward connections (grey lines). **(B)**. Test accuracy after one training epoch on the MNIST dataset for networks with different numbers of hidden layer nodes and connection probabilities between nodes in the hidden layer when nodes use the hyperbolic tangent activation function instead of ReLU.

We next investigated how this finding depends on other network properties. We find that the advantage conferred by sparse connectivity is limited to networks with a recurrent architecture (Figure 3A). In feedforward networks, sparse connectivity has very little effect on the performance of large networks, and is typically detrimental in small networks (Supplementary Figure S3). We had so far constructed networks using the ReLU activation function (see Supplementary Figure S1), with which nodes require a threshold of net excitation received before any output is produced. This is reminiscent of neuronal integration, whereby neurons require net excitatory synaptic input to surpass a certain threshold before an action potential response is elicited, and otherwise produce no output. We therefore next tested whether our finding still holds when using an activation function whose properties are very different from neuronal integration, e.g., the symmetrical hyperbolic tangent (tanh) function, which can produce positive and negative outputs. We find that with this less biologically realistic activation function, sparse connectivity no longer improves learning efficiency in large networks (Figure 3B). The tanh activation function has been reported to cause vanishing gradients (Ven and Lederer, 2021). To investigate whether this may explain our results, we repeated training and recorded the gradients during the first epoch of training in large sparse networks (10,000 hidden layer nodes, connection probability 0.1). Surprisingly, networks with the tanh activation function have slightly larger gradients than those with the ReLU activation function (mean gradient magnitude 2.0×10^−5^ for tanh network and 6.0×10^−6^ for ReLU network), indicating that the failure of large networks using tanh is not simply due to vanishing gradients.

**Figure 4:**
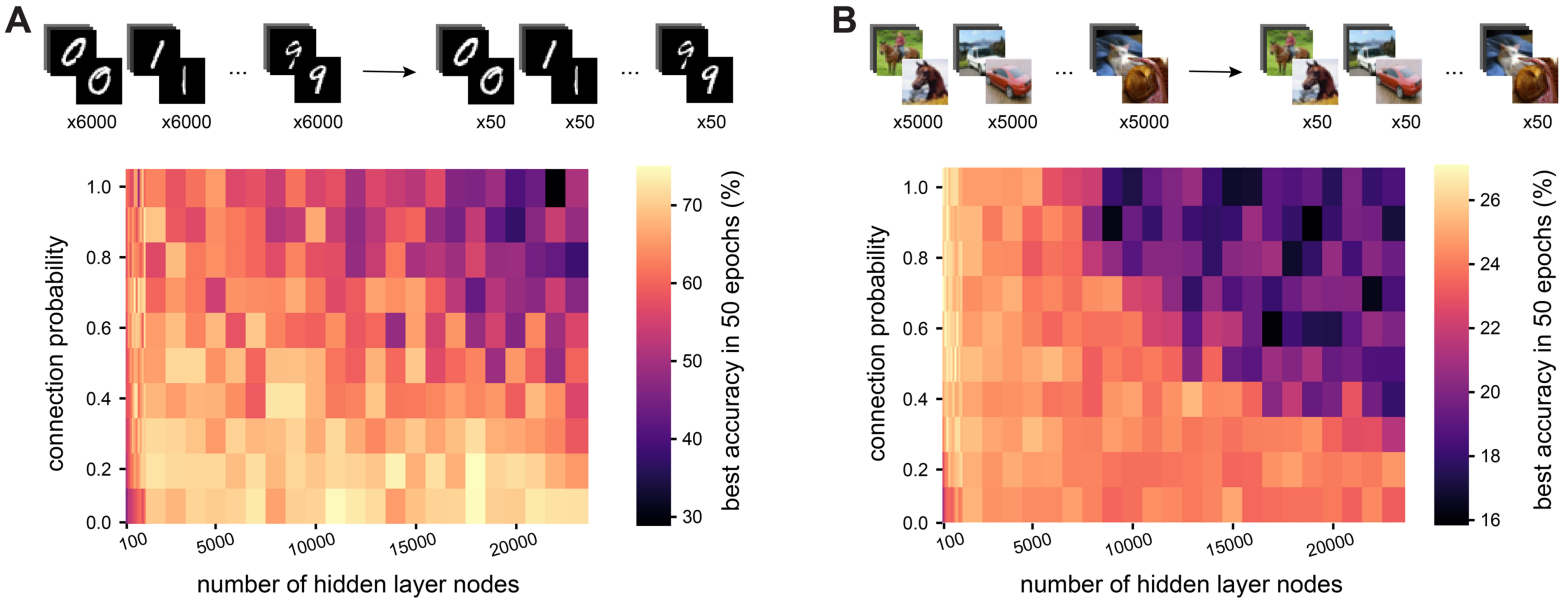
Sparse connectivity enables data-efficient training of large and recurrent networks. **(A)**. Test accuracy after one training epoch on a reduced version of the MNIST dataset (50 examples per class instead of 6,000) for networks with different numbers of hidden layer nodes and connection probabilities between nodes in the hidden layer. **(B).** Test accuracy after one training epoch on a reduced version of the CIFAR10 dataset (50 examples per class instead of 5,000) for networks with different numbers of hidden layer nodes and connection probabilities between nodes in the hidden layer.

Having established that sparse connectivity can improve network performance in large and recurrent networks when training time is limited, we next investigated whether the same is true with data limitations. To this end, we trained networks on a reduced training set from the MNIST dataset, where only a subset of samples from each class was used during training (50, 100, 500 or 1,000 samples per class – results for 100, 500 and 1,000 samples per class, as well as for the full dataset are found in Supplementary Figure S4). The test dataset remained unaltered. Training time was not a constraint in these experiments, so all networks were trained for 50 epochs and we recorded the best test accuracy attained during this training period. We find that in large (20,000 hidden nodes) recurrent networks, sparse connectivity becomes more advantageous the more restricted training data are: while on the full training dataset of 60,000 examples, sparse and dense networks attain comparable maximum test accuracies (99.0 ± 0.1% vs. 98.5 ± 0.1%), their performance diverges the more restricted the training dataset is, with respective test accuracies of 70.1 ± 10.2% and 40.3 ± 7.2% when trained on only 500 examples (50 per class, Figure 4A). Meanwhile, in small networks (500 hidden nodes), we again see that sparse connectivity is detrimental to the training process, with test accuracies only reaching 57.0 ± 1.3% in sparse networks but 66.0 ± 4.0% in dense networks when trained on the most restricted training dataset. Repeating this experiment on the CIFAR10 dataset, large sparse networks outperform their dense counterparts on all subsets of the training data, with test accuracies of 54.9 ± 0.1% vs. 42.1 ± 1.8% on the full training dataset, and 24.0 ± 0.1% vs. 17.6 ± 1.5% on the most restricted dataset (Figure 4B). Our findings therefore suggest that in large and recurrent networks, as found in the cortex, sparse connectivity enables the network to learn efficiently in terms of both training time and training data.

### Sparsely connected networks form distributed, robust representations

**Figure 5:**
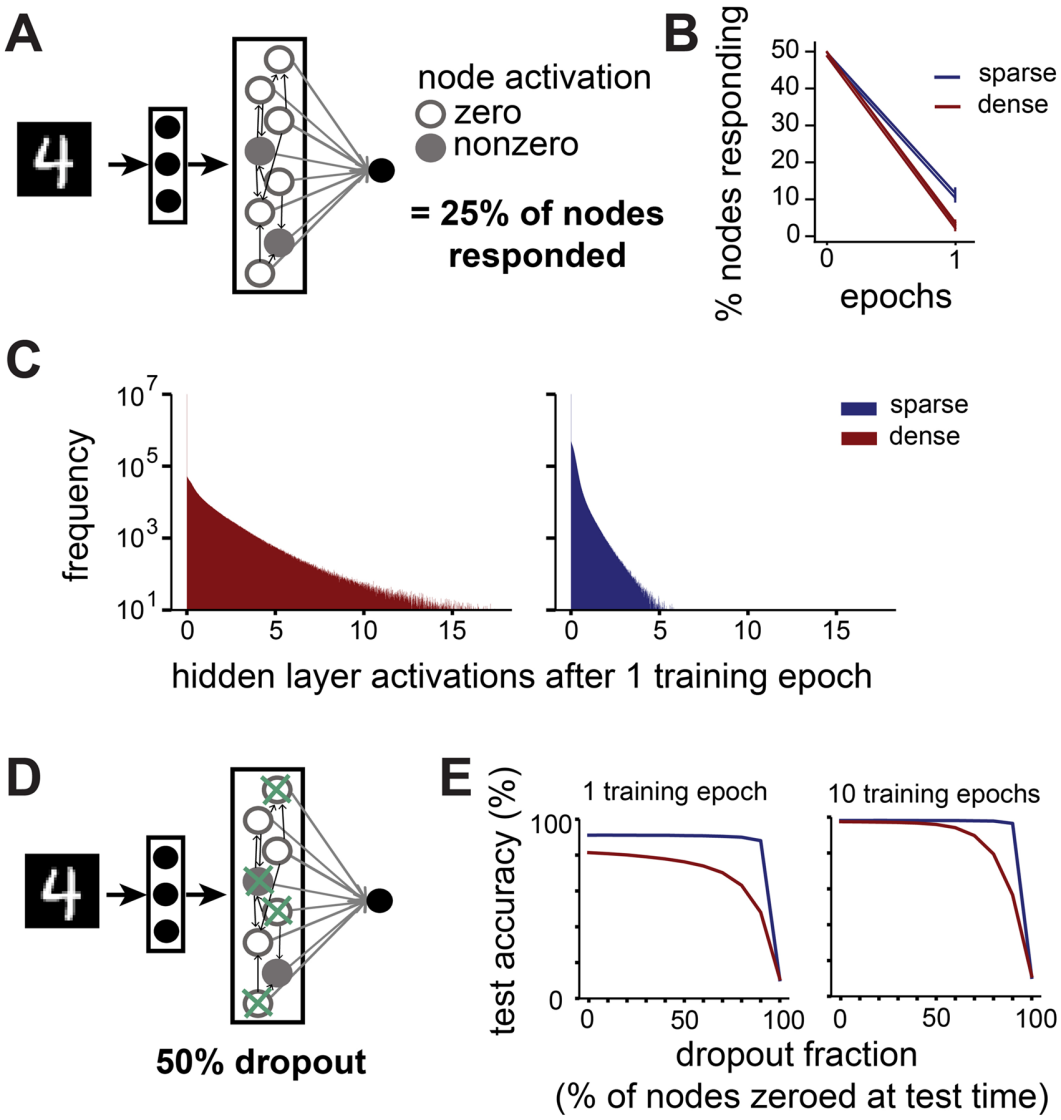
Sparsely connected networks form distributed, robust representations. **(A)**. We recorded how many nodes in the hidden layer send zero and nonzero activations to the output layer in response to all images from the MNIST test dataset (*N* = 10,000). **(B)**. Change in percentage of nodes sending a nonzero activation to the output layer in the first training epoch on MNIST. **(C)**. Distribution of activation values sent from hidden layer nodes to the output layer by large networks (10,000 hidden layer nodes) with dense (connection probability = 1, red) or sparse (connection probability = 0.1, blue) connectivity in response to all images from the MNIST test dataset after one training epoch. **(D)**. We performed a dropout experiment where the output from a randomly selected fraction of nodes in the hidden layer was set to zero at test time. **(E)**. Effect of different dropout fractions on test accuracy in large networks (10,000 hidden layer nodes) with dense (connection probability = 1, red) or sparse (connection probability = 0.1, blue) connectivity after 1 training epoch (left) and 10 training epochs (right).

To determine how inputs are represented by networks with different connectivity properties, we recorded the activation values of nodes in the hidden layer when presented with all examples from the test dataset. To assess whether activity is sparse or dense, we first recorded whether a node produces a zero or non-zero activation in the final timestep, which is passed to the output layer (Figure 5A). We find that in large networks (10,000 hidden nodes), already after a single training epoch inputs are sparsely represented by the hidden layer activity in both sparsely and densely connected networks, with a majority of hidden layer nodes sending zero-activations to the output layer in response to any given image (Figure 5B). Surprisingly, we find that in sparsely connected networks more nodes contribute non-zero outputs to the classification than in densely connected networks (11.2 ± 1.5% vs. 3.0 ± 1.0% after one training epoch). When looking at the activation values, not just whether they are zero or non-zero, we note that the average magnitude of hidden layer activations is larger in densely connected networks and smaller in sparsely connected networks (Figure 5C). We calculated the mutual information between the outputs of 10,000 randomly chosen pairs of hidden layer nodes for a sparse and a dense network. We find that the mutual information is significantly higher between nodes in the sparsely connected network (mean 0.019 ± 0.065) compared to densely connected networks (mean 0.005 ± 0.029, KS-test *p* = 1.03×10^−44^). A larger proportion of nodes contributing to the classification with smaller activations suggests that large sparse networks employ a more distributed, consensus-based coding strategy than dense networks.

Such a distributed code may have advantages in terms of robustness to noise, as the output of any individual node may be less important for the final classification. To test this, we set the output to the classification layer of a proportion of randomly selected nodes to zero for each image at test time and assessed the networks’ performance (Figure 5D). Indeed, we find that sparsely connected networks outperform their dense counterparts at all noise levels (Figure 5E). For instance, when 50% of all nodes’ outputs are set to zero after one epoch of training, the test performance of sparse networks is barely affected (from 91.1 ± 0.4% to 90.9 ± 0.6%), while the performance of dense networks suffers more (from 81.4 ± 4.6% to 76.3 ± 5.8%). This discrepancy is even more noticeable at higher dropout levels, with sparse networks’ performance only dropping by 3.1%, while dense networks lose 33.3% in accuracy when 90% of nodes are zeroed out. With more training, dense networks gradually become more robust to low levels of dropout noise, but even after 10 training epochs they are still outperformed by sparse networks at high dropout levels.

### Sparse connectivity facilitates efficient training in networks with fixed excitatory and inhibitory nodes

**Figure 6:**
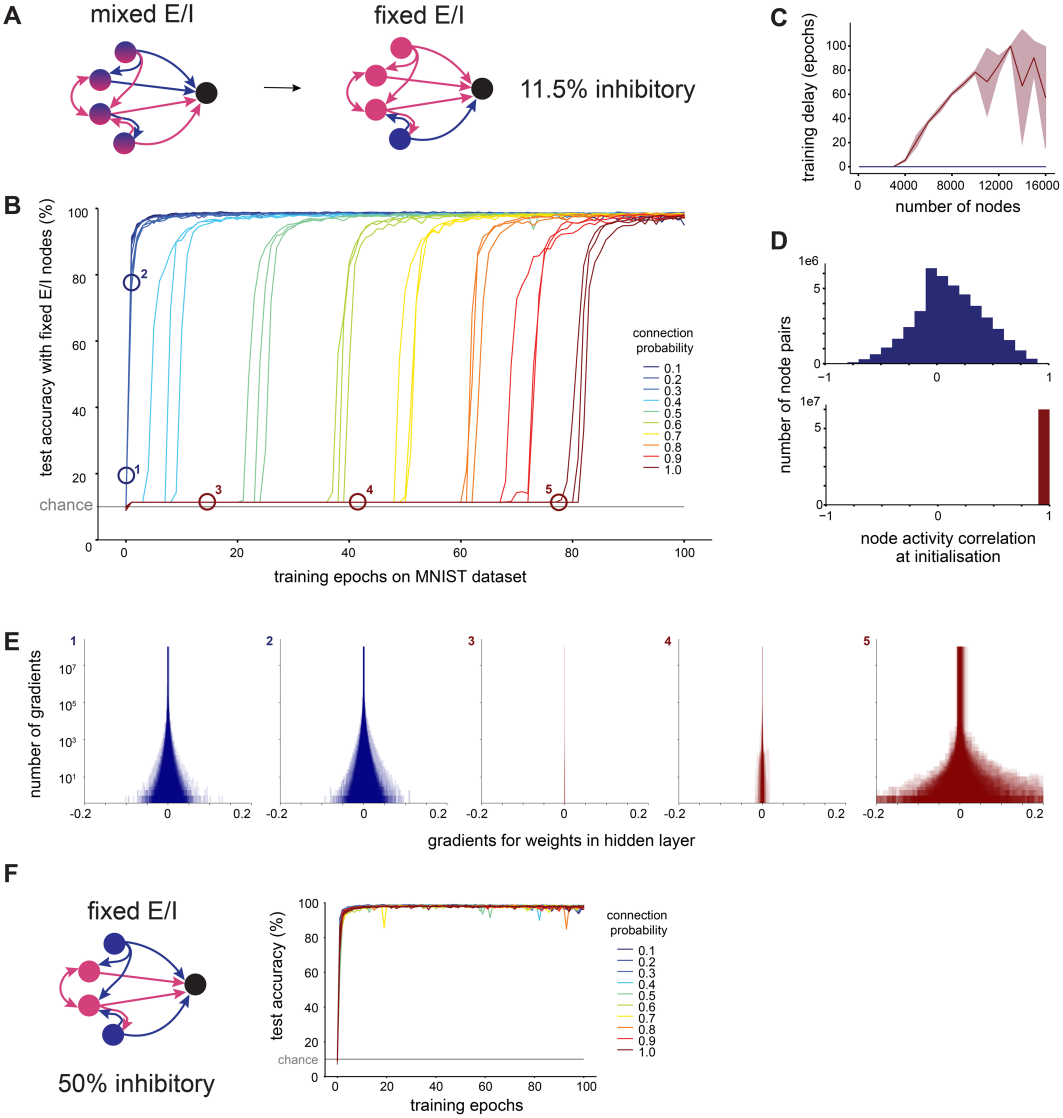
Sparse connectivity facilitates efficient training in networks with structural E/I imbalance seen in cortex. **(A)**. We constructed recurrent ANNs where each node was fixed to either excitatory (only positive outgoing weights) or inhibitory (only negative outgoing weights). We set 11.5% of nodes to be inhibitory, as reported in measurements from somatosensory cortex. **(B)**. Test accuracy over time in large networks (10,000 hidden layer nodes) trained on MNIST with fixed excitation and inhibition and different hidden layer connection probabilities. **(C)**. Training delay (number of epochs before the network’s performance exceeds chance level) as a function of the number of nodes in the network’s hidden layer, for dense (connection probability = 1, red) and sparse (connection probability = 0.1, blue) connectivity, shaded area shows standard deviation. **(D)**. Distribution of Pearson’s R correlation coefficients between hidden layer node activations in response to all MNIST test dataset images, at initialization (before any training), for dense (connection probability = 1, red) and sparse (connection probability = 0.1, blue) connectivity with 10,000 hidden layer nodes. **(E)**. The gradient of a weight represents the rate of change of the loss function with respect to that weight, providing a measure of how modifying the weight will influence the network’s overall error. We visualize distributions of gradients for hidden layer weights for sparse (connection probability = 0.1, blue) and dense (connection probability = 1, red) networks with 10,000 hidden layer nodes. Numbers correspond to labels in panel B. **(F)**. We repeated the same experiment with fixed excitatory and inhibitory nodes, but now set 50% of nodes to be inhibitory (balanced E/I). Test accuracy over time is shown for networks with 10,000 hidden layer nodes.

How do these sparse representations form during training? When recording the changes in hidden layer weights during training, we observe a greater tendency for negative weights to increase their magnitude, and positive weights to change their sign and become negative, than vice-versa (Supplementary Figure S5A). This leads to a sparse representation as the weighted sum of inputs to any given node is more likely to be negative, resulting in an output of zero after the ReLU activation function is applied. This extent of sign reversal of weights is biologically implausible, as each neuron generally transmits the same set of neurotransmitters to all of its post-synaptic partners, and cannot change this for individual connections (Dale’s principle). Therefore, we constructed and trained networks which obey Dale’s principle, with fixed excitatory and inhibitory nodes, reminiscent of cortical neuronal cell types (Figure 6A). Note that while the clear distinction of neurons into excitatory and inhibitory is valid in cortex, this may not be true for all neurons [e.g., neuromodulatory neurons in the cholinergic system (Saunders et al., 2015)]. We chose the proportion of inhibitory nodes which has been reported experimentally for somatosensory cortex (Meyer et al., 2011) (11.5% inhibitory, 88.5% excitatory). Inhibitory nodes were initialized with random, all negative outgoing weights, and excitatory nodes with random, positive outgoing weights. Inhibitory and excitatory weights’ magnitudes were sampled from the same uniform distribution, and the connection probability was applied in the same way to all connections, regardless of whether they were excitatory or inhibitory. During training, if any weight’s sign would be reversed, its value was set to zero instead.

In line with reports from others, we note that the training of large (10,000 nodes), densely connected networks is indeed severely impeded by the constraints of Dale’s principle (Figure 6B). We observe a delay of tens of training epochs at the start of the training process, during which the network’s performance remains around chance level. However, the sparser the connectivity, the shorter the delay before a network’s performance shows improvement. The length of this delay in training of densely connected networks increases with the size of the network, and no delay was observed at any tested network size for sparsely connected networks with a connection probability of 0.1 (Figure 6C).

We investigated why sparse networks outperform their dense counterparts under these conditions. When examining the activations sent from the hidden layer to the output layer, we find that at initialization, activations from the hidden layer nodes of densely connected networks are highly correlated with each other, whereas those from sparsely connected networks are less correlated (Figure 6D). This implies that in densely connected networks, all nodes initially produce very similar outputs, which would make it more challenging to develop meaningful, distinguishable outputs through training.

The gradient of a weight represents the rate of change of the loss function with respect to that weight, providing a measure of how modifying the weight will influence the network’s overall error. Therefore, recording the gradients during training allows us to observe the shape of the error landscape. We find that during early training epochs (with the exception of the first epoch), densely connected networks have very small gradients associated with their weights, while the gradients in sparsely connected networks are much larger (Figure 6E). This suggests that densely connected networks, unlike their sparse counterparts, become stuck on a plateau in the error landscape, i.e., a region with a high error and small gradients. This plateau is difficult to leave via gradient descent, and therefore causes a delay in learning.

To test the hypothesis that this is indeed a feature of the weight initialization, and not some other aspect of training, we initialized a network with a sparse weight matrix (i.e., with 90% of weights starting at zero), but then allowed all weights to be modified during training regardless of their starting value, as would be the case for a dense network. These “sparse-to-dense” networks all start training without a delay like standard sparse networks, but their learning rate soon slows and they take longer to reach their peak performance, similar to networks with more dense connectivity (Supplementary Figure S5B).

The hidden layer activations in densely connected networks with a learning delay were not only highly correlated, but also very large, which led us to speculate that these networks may be experiencing an excitation-inhibition (E-I) imbalance due to the low proportion of inhibitory nodes. To test this, we repeated the training of Dale-compliant networks with a biologically unrealistic, balanced proportion of excitatory and inhibitory nodes (50% excitatory, 50% inhibitory). We found that these balanced networks did not experience a delay in training, regardless of their density (Figure 6F). Similarly, when we initialise networks with the imbalance of excitatory and inhibitory nodes found in cortex as before (11.5% inhibitory, 88.5% excitatory), but increase the initial magnitude of inhibitory weights ten-fold, there are no delays in training, regardless of the network’s density (Supplementary Figure S5C). These findings confirm that densely connected networks with a biologically plausible fraction of inhibitory nodes experience an E-I imbalance which prevents them from learning efficiently. We show that this imbalance can be partly mitigated by sparse connectivity.

## Discussion

We generated and trained ANNs constrained by interpretable features of cortical networks in order to disentangle the effect of structural properties on network function. We find that sparse connectivity is a prerequisite for efficient learning when the network adheres to certain other properties of cortical networks: large recurrent networks, even more so when nodes are either excitatory or inhibitory like cortical neurons. For biological context, the smallest computational unit of the cortex is often reported to be the cortical column, consisting of 10,000–20,000 neurons, with connectivity between 10–30% (Meyer et al., 2013). Therefore, the large and sparse networks we investigated here share the same parameters as this elementary computational unit of the cerebral cortex.

From a machine learning perspective, many attempts have been made to achieve high performance with sparse networks due to their potential for lower computational and memory demands. A significant approach to generating performant sparse networks is pruning, which begins by training a densely connected network and then removing (pruning) edges which are deemed unimportant throughout the training process, to eventually obtain a sparse network. Pruning approaches are able to generate sparse networks whose performance does not significantly differ from dense networks for both feedforward (Han et al., 2015) and recurrent (Narang et al., 2017) networks, but still requires starting with a densely connected network. A related method is rewiring, which begins with a sparse network, and then allows existing edges to be pruned and new edges to be formed during training, while maintaining a constant total number of edges. This was shown to produce performance equivalent to densely connected networks for feedforward architectures (Dettmers and Zettlemoyer, 2019), and in fact resulted in sparse networks which outperformed their dense counterparts for recurrent architectures (Liu et al., 2021). Here we show that, given the right conditions, a sparse recurrent network can outperform its dense counterparts even with fixed sparse connectivity.

Given the extent of non-random wiring observed in the cortex (Song et al., 2005; Udvary et al., 2022), it is somewhat surprising that sparse networks with random connectivity perform as well as they do here. There is evidence for random connectivity in some biological neural networks, like olfactory inputs to the mushroom body in *Drosophila* (Caron et al., 2013). This aligns with computational models like the Liquid State Machine, which posits that a sufficiently large population of randomly interconnected neurons can generate a diverse enough set of input representations, even in the absence of learning, for a simple downstream classifier to learn to distinguish between inputs (Maass, 2011). There is some evidence that the same may be true in ANNs: Frankle and Carbin (2019) find that sparse subnetworks exist in randomly initialised densely connected ANNs which, when trained in isolation, achieve at least equivalent performance to the full-sized dense network with less training. This finding suggests that within large, randomly initialised networks, there exist structures which are inherently well-suited for learning and/or performing the task at hand, which could explain why our large, sparse networks perform well even with randomly initialised connectivity. It may be interesting to investigate even larger networks, perhaps mimicking other neuronal structures, e.g., the cerebellum, to determine whether this property continues to hold true. A systematic evaluation of the relevance of non-random connectivity remains an open topic for future investigation.

Our results suggest that the integrative properties of nodes (i.e., their activation function) are relevant for determining whether a network will benefit from sparse connectivity. However, a linearly weighted sum followed by a ReLU activation function by no means reproduces the complex input–output computations performed by real neurons. For example, unlike the nodes in our ANNs, synaptic conductance and therefore the activation of biological neurons are stochastic (Rusakov et al., 2020). There are strong parallels between stochastic activity and the practice of node dropout during training in machine learning, as both result in the absence of activity from a varying subset of neurons/nodes. Dropout has been shown to reduce overfitting (Srivastava et al., 2014), a problem which we observed in our large, sparse networks on the CIFAR10 dataset. This suggests that incorporating additional biological details on a single-cell scale may also be fruitful.

Sparsely and densely connected networks differ in their node activity, and therefore in their representations of input. We found that in sparsely connected networks, more nodes participate in the classification than in densely connected networks. We have previously shown that correlations in in-degrees (i.e., correlations between the number of synaptic inputs received by a neuron from excitatory and inhibitory presynaptic populations) are a mechanism to compensate for heterogeneous inputs and enable balanced state dynamics, where a majority of nodes are able to contribute to signal processing (Landau et al., 2016). Here, we show that sparse connectivity could be another way to facilitate a balanced state and reduce quiescence. However, by itself sparsity is insufficient to account for the broad representation typically seen experimentally in cortical recordings. This indicates that higher-order features of connectivity like degree correlations are a key target for future investigation. In addition to degree correlations, biological neural networks may make use of specific synaptic plasticity mechanisms in order to equalize excitation-inhibition ratios and thereby regulate the activity of individual neurons (Xue et al., 2014). We used global backpropagation of error and gradient descent-based optimisation to train our networks, which cannot account for local synaptic plasticity mechanisms. The impact of more local learning rules should therefore also be the topic of further studies.

Why do sparsely connected networks perform better than densely connected networks under these conditions? Our analysis of node activations in Dale’s networks shows that the outputs of nodes in densely, but not sparsely connected networks are highly correlated at initialisation, a relationship which has also been reported in spiking neural networks (Pernice et al., 2011). We find that this is likely due to an excitation-inhibition imbalance here, whose effects are mitigated by sparse connectivity. Correlations in activity between neurons in biological neural networks are thought to be relevant for information processing (Shadlen and Newsome, 1998; Averbeck et al., 2006) and learning (Bi and Poo, 2001). However, the extremely high correlations in our densely connected networks suggest a high redundancy in information across nodes, and therefore a very limited effective capacity of the network. This could make it more difficult for the dense network to develop distinguishable representations of its inputs, and thereby explain the delay in learning. Our finding that gradients in dense networks are very small during the delay in training supports this hypothesis, but there is a need for methods to better characterize the error landscape.

It has been speculated that innate behavioral abilities and rapid learning in animals may be facilitated by specific wiring properties in neural circuits that emerge during development (Zador, 2019). As the information capacity of the genome is orders of magnitude too small to encode the connection between each pair of neurons explicitly, it was suggested that wiring rules may underlie the formation of neural circuits in the developing brain. It is indeed plausible that such wiring rules could inform biological neural networks, as exemplified by a study proposing rules connecting different innexins to form gap junctions in the nervous system of *C. elegans* (Kovács et al., 2020), or another showing that just the structural composition of the neuropil is sufficient to explain a large portion of the non-random connectivity observed in the rat somatosensory cortex (Udvary et al., 2022). To further support this, we here find that even a ‘wiring rule’ as simple as sparse connectivity can facilitate efficient information processing.

In summary, we show that sparse connectivity enables efficient information processing given some key features of cortical networks, and set the stage for the investigation of a variety of other features of biological networks.

## Methods

### Code availability

All code needed to reproduce the results in this study can be found at: https://github.com/mpinb/sparseANNs.

We systematically investigated the effect of size and sparse connectivity on the training and performance of recurrent ANNs. To this end, we generated a range of ANNs with different numbers of nodes in the hidden layer, and with different connection probabilities between the hidden layer nodes. All nodes were arranged in a single hidden layer, and any node in the hidden layer could be connected to any other node, with the number of such connections determined randomly by the connection probability. The lower the connection probability the sparser the network, i.e., the fewer nodes were connected to each other by a trainable weight. Formally, the density of a network is given as D = E / [N * (N-1)], where E is the number of edges in the network, and N is the number of nodes. The network density in the hidden layer of our networks is therefore functionally equivalent to the connection probability, as the connection probability is applied to all edges in the hidden layer. To confirm this, we calculated the network density as above for all 10 networks with 10,000 hidden layer nodes and connection probability of 0.1, and all were equivalent to within 3 significant figures. The weights of edges between hidden layer nodes were randomly initialised from a uniform distribution between −0.001 and 0.001, with weights between unconnected nodes fixed at zero throughout. For each set of hyperparameters (network size & sparsity) we trained 10 networks with different random initialisations, which differ in their connectivity matrix (specifying which nodes are connected by a trainable weight) and in the values of edge weights at initialisation. Results are given as mean ± standard deviation unless otherwise specified. All nodes in our ANNs use the rectified linear unit (ReLU) activation function unless otherwise specified.

The primary limitation to network size in this study was hardware constraints, specifically the VRAM available to store the network on the GPU for training. We trained most of our networks on NVIDIA Quadro RTX 6000 GPUs, which have 24GB of VRAM. The largest networks (24,000 hidden layer nodes and larger from Figure 2), were trained on NVIDIA A100 GPUs, which have 80GB of VRAM, as they were too large to fit into VRAM on the other GPUs.

We evaluated our networks on three benchmark machine learning tasks, MNIST (Lecun et al., 1998) and CIFAR10 (Krizhevsky, 2012) image classification, and Sleep-EDF (Kemp et al., 2000) sleep-stage classification from EEG recordings. The image-based tasks were modified for recurrent networks to encode each image as a time series by slicing each image row-wise and presenting one row of pixel values (one value per pixel for grayscale images in MNIST, three values per pixel for RGB images in CIFAR10) in each time step (Supplementary Figure S1B). The size of the input layer was equal to the width of the image (MNIST, 28), the width of the image multiplied by 3 to account for RGB color channels (CIFAR10, 32×3), or one for the single-channel timeseries in the Sleep-EDF dataset. Each input layer node was connected to all hidden layer nodes by a trainable weight. The output from all the recurrently connected hidden layer nodes in the last time step was then passed to a linear output layer with 5 (Sleep-EDF) or 10 (MNIST & CIFAR10) nodes for classification (one for each class in the dataset, target classes were one-hot encoded). Networks were trained by backpropagation using the ADAM optimiser for gradient descent, with a learning rate of 0.001. We used the cross entropy loss function to evaluate the performance of ANNs during training. All networks were implemented and trained in PyTorch v1.8.1.

To assess the single-epoch learning performance of our networks, we trained all networks for one epoch, meaning that each data point from the training dataset was presented once (60,000 data points for MNIST, 50000 data points for CIFAR10, 398,370 data points for Sleep-EDF). Then, the accuracy of the networks was tested on the corresponding testing dataset, which consists of 10,000 (MNIST & CIFAR10) or 170,730 (Sleep-EDF) unseen examples.

To assess whether recurrent network architecture was necessary for sparsity to be beneficial, we repeated the same training of networks with different hyperparameters with feedforward networks. Here, hidden layer nodes were split into two hidden layers. Nodes in the first layer could be connected to nodes in the second hidden layer by a feedforward connection, with the number of such connections determined randomly by the connection probability. To feedforward networks, the whole image was provided at once as input (flattened to a 1D vector) for MNIST and CIFAR10 datasets, and for the Sleep-EDF dataset, the whole EEG sequence was passed at once. The length of the resulting input vector determined the size of the input layer.

To evaluate the effect of a different activation function, we replaced the ReLU activation function in hidden layer nodes with the hyperbolic tangent (tanh) function. All other hyperparameters remained unchanged, and we trained three different random initialisations for each set of hyperparameters with this new activation function. The tanh activation function has been reported to cause vanishing gradients (Ven and Lederer, 2021). To investigate this, we repeated training and recorded the gradients during the first epoch of training in large sparse networks (10,000 hidden layer nodes, connection probability 0.1).

To assess the performance of our networks with limited training data, we modified the MNIST and CIFAR10 tasks. The full training datasets contain 6000/5000 examples of each class for MNIST and CIFAR10, respectively. We selected a smaller random subset of examples (50, 100, 500 or 1,000 per class) and used only these for training. The test dataset remained unchanged. ANNs were trained for 50 epochs with early stopping if their performance stopped improving, and we then compared their best test accuracy.

In order to investigate how our networks encode their inputs, we determined how hidden layer nodes contribute to a classification. To this end, we took a sparsely connected network (size 10,000 hidden nodes, connection probability 0.1) and a densely connected network (size 10,000 hidden nodes, connection probability 1.0). Then, we gave each network the full testing dataset of MNIST (10,000 images) as input. After the network had processed each image, we recorded the activation values (outputs) of all hidden layer nodes at the last time step, which is the activation value which is passed to the output layer for classification. We performed these measurements before the networks had received any training, and after one epoch of training. We first consider what proportion of hidden layer nodes send a non-zero activation to the output layer in response to each image. Then, we also consider the magnitude of these non-zero activations. We used sklearn.feature_selection.mutual_info_regression from scikitlearn (Pedregosa et al., 2011) to calculate the mutual information between the responses to the whole testing dataset for 10,000 randomly sampled node pairs.

Finally, we constructed ANNs which obey Dale’s principle (Eccles, 1976), meaning that each node had either exclusively positive or negative outgoing weights, and this sign remained unchanged throughout the training process. We initialised networks with 11.5% inhibitory nodes, corresponding to the proportion of inhibitory neurons reported in sensory cortex (Meyer et al., 2011). Inhibitory nodes were initialised with random, all negative outgoing weights, and excitatory nodes with random, positive outgoing weights (all weights still had a magnitude randomly chosen according to a uniform distribution between 0 and 0.001). Whether a node had a trainable connection to another node was purely determined by the connection probability, and was not affected by their excitatory or inhibitory nature. After each weight update, if any weight’s sign would be reversed, its value was set to zero instead. All other training parameters were the same as for our other networks. Exceptions are several manipulations which we performed on these networks: the “sparse to dense” networks in Supplementary Figure S5B had the network initialised the same way as a sparse network with a connection probability of 0.1, but then weights initialised at zero were allowed to become nonzero during training. For Supplementary Figure S5C, we initialised and trained the networks as before, with 11.5% inhibitory nodes, except that we initialised the outgoing weights from inhibitory nodes with a 10x larger magnitude than excitatory weights. For Figure 6F, we initialised networks with 50% inhibitory nodes to evaluate the effect of structural E/I balance.

## Data Availability

Publicly available datasets were analyzed in this study. This data can be found at: https://github.com/mpinb/sparseANNs.
