# Supplementary material for: Sparse connectivity enables efficient information processing in cortex-like artificial neural networks
Source: Front Neural Circuits. 2025 Mar 13;19:1528309. doi: 10.3389/fncir.2025.1528309 (PMC11966417; doi:10.3389/fncir.2025.1528309)
Supplement: Supplementary file 4 [file Data_Sheet_1.docx]

## Supplementary Materials

## Supplementary Figures


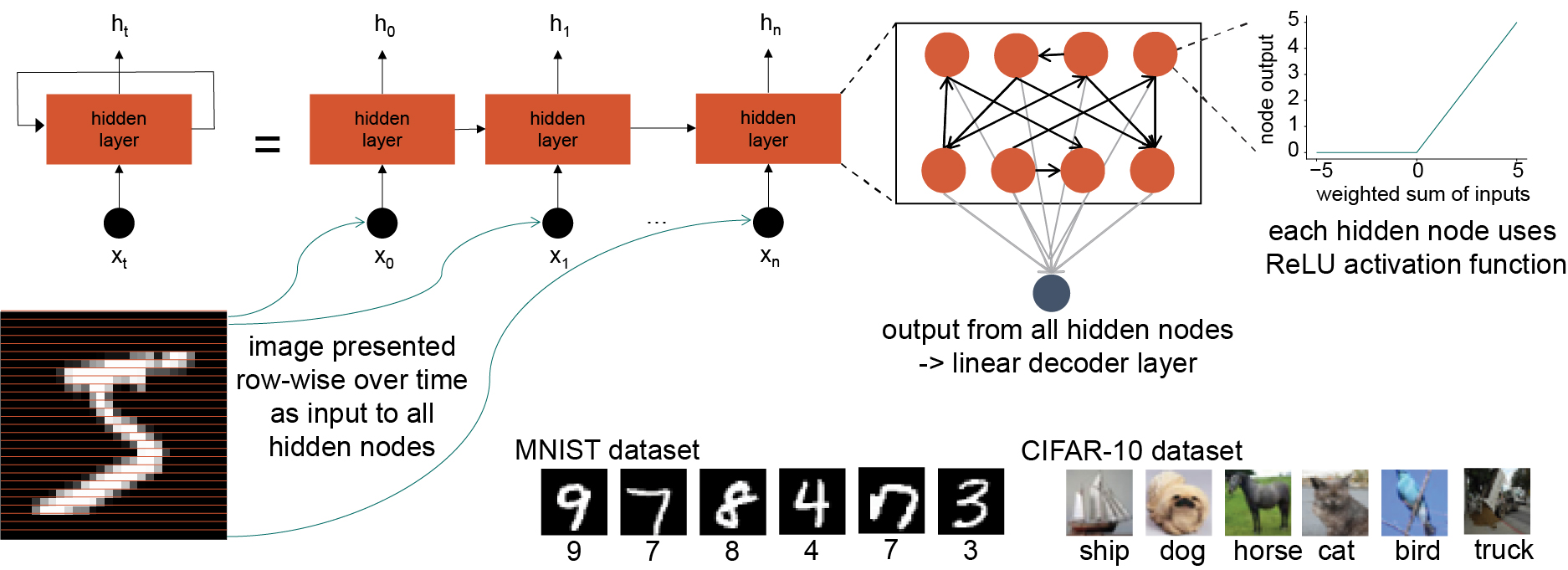


**Fig. S1.** Schematic illustration of ANNs. Sparsely connected recurrent: nodes in the recurrent hidden layer are arranged into a single layer. Within this recurrent layer, any node can be connected to any other node with a trainable weight. The number of connections changes according to the connection probability. Each node uses the ReLU activation function. The input and output layers are fully connected to all nodes in the hidden layer. We modify image classification tasks (MNIST & CIFAR10) for use with recurrent networks. The image was split row-wise, and at each time step the next row of the image was provided as input to all nodes in the recurrent layer.


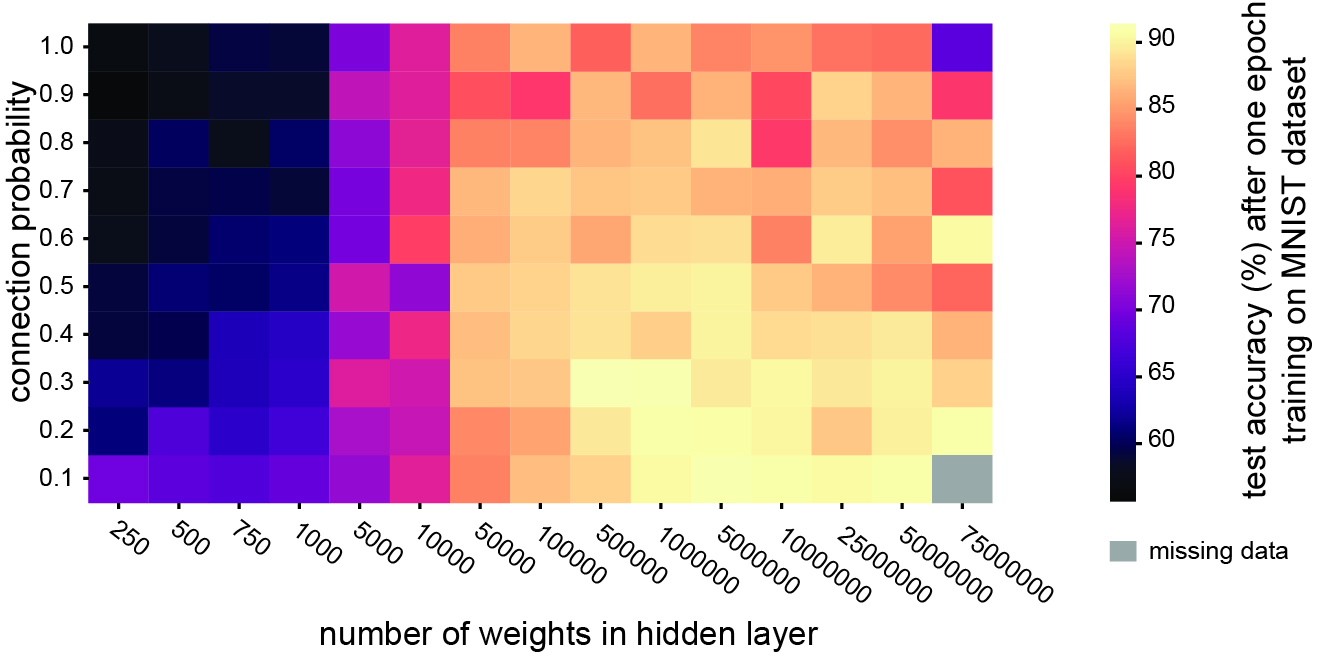


**Fig. S2. Sparsity is beneficial even when the number of hidden layer weights is kept constant.** Test accuracy after one training epoch on the MNIST dataset for recurrent networks with the same number of weights in the hidden layer, while systematically varying the connection probability between nodes in the hidden layer.


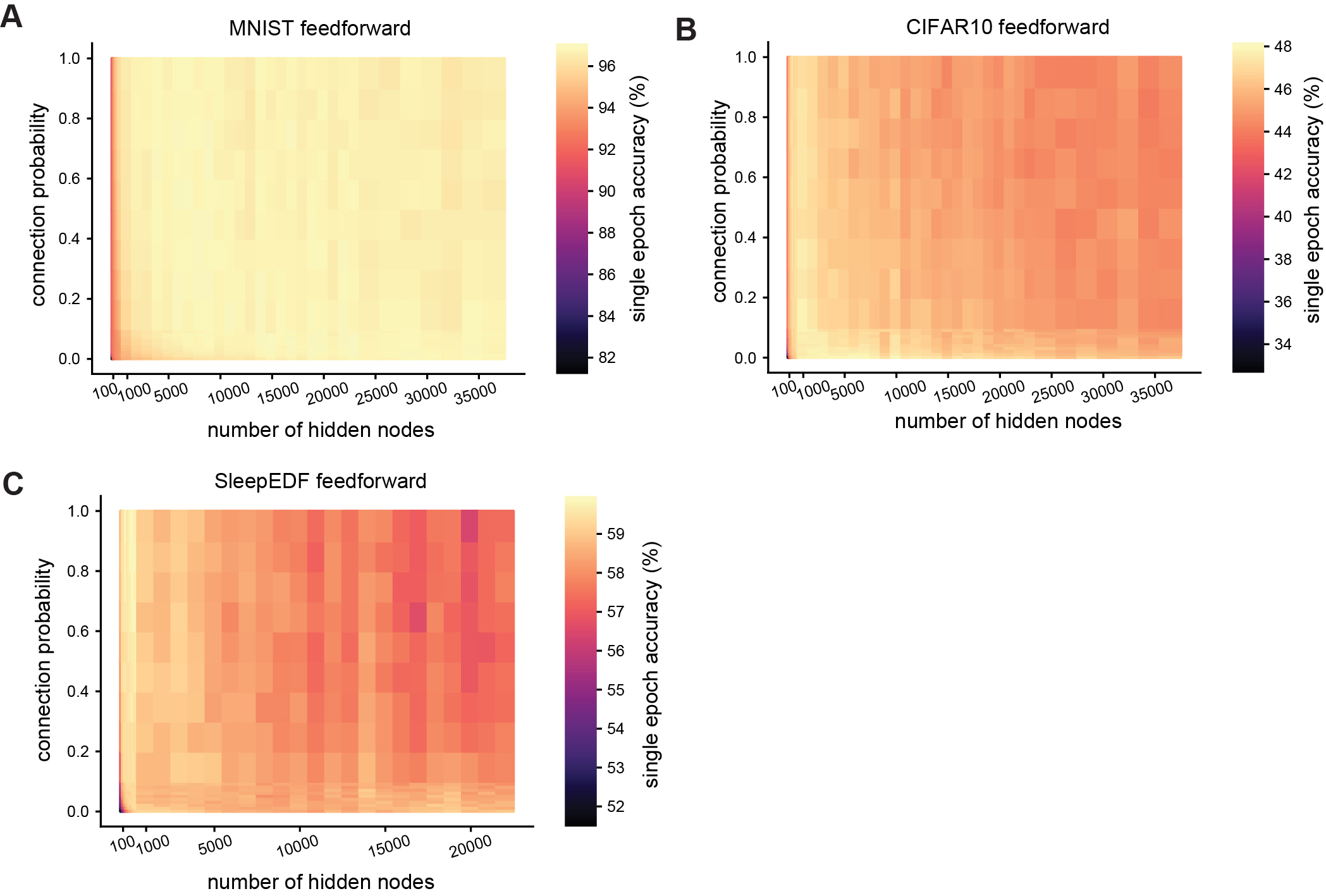


**Fig. S3. Sparsity confers minimal benefit in feedforward networks. A.** Test accuracy after one training epoch on the MNIST dataset for feedforward networks with different numbers of hidden layer nodes and connection probabilities between nodes in the hidden layers. **B.** Test accuracy after one training epoch on the CIFAR10 dataset for feedforward networks with different numbers of hidden layer nodes and connection probabilities between nodes in the hidden layers. **C.** Test accuracy after one training epoch on the Sleep-EDF dataset for feedforward networks with different numbers of hidden layer nodes and connection probabilities between nodes in the hidden layers.


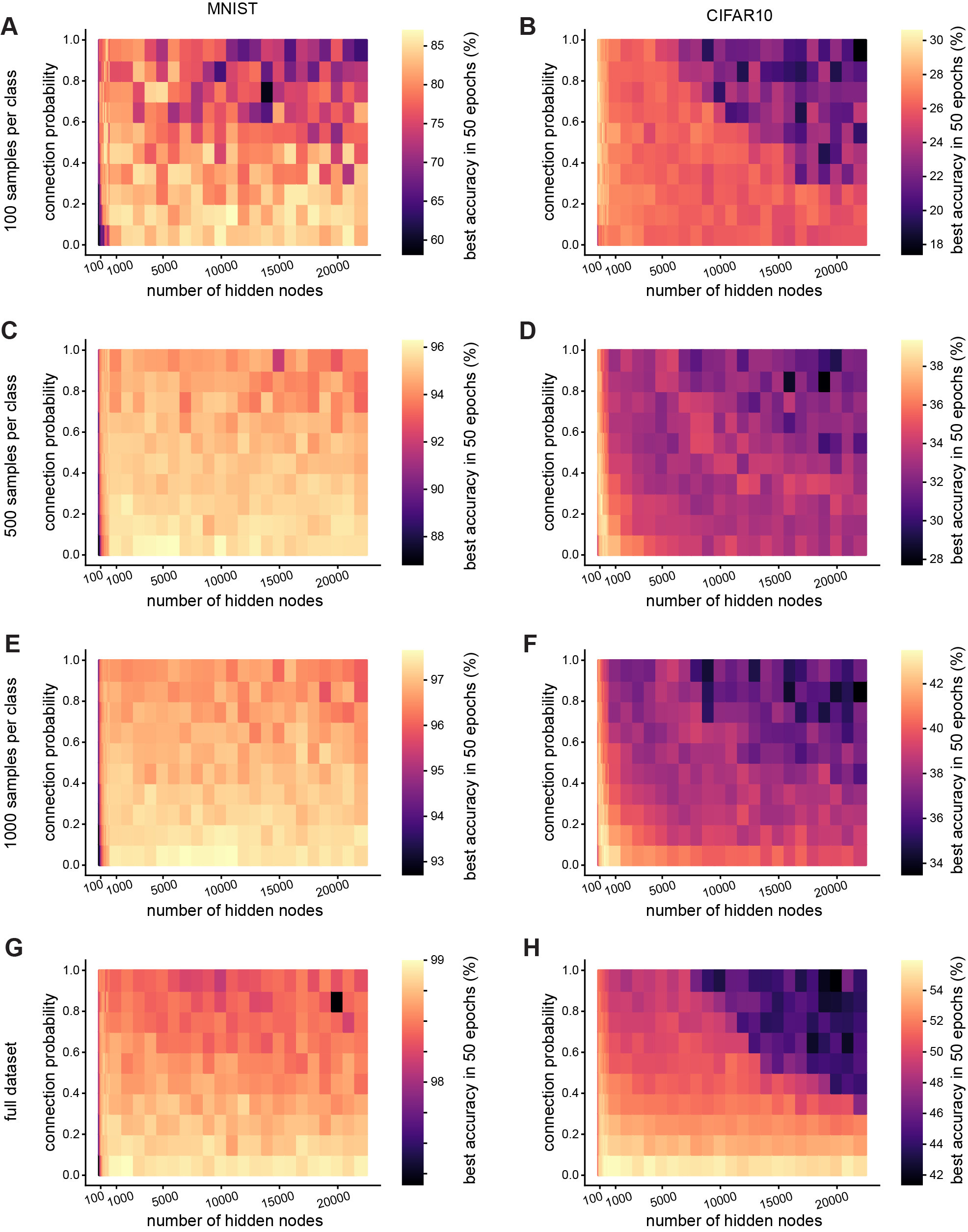


**Fig. S4. Reduced training data supplement. A-H.** Test accuracy after one training epoch on for recurrent networks with different numbers of hidden layer nodes and connection probabilities between nodes in the hidden layers. Left column: MNIST dataset, right column: CIFAR10 dataset. Rows top to bottom: training dataset reduced to 100 examples per class, training dataset reduced to 500 examples per class, training dataset reduced to 1000 examples per class, full training dataset (6000 examples per class for MNIST, 5000 examples per class for CIFAR10).


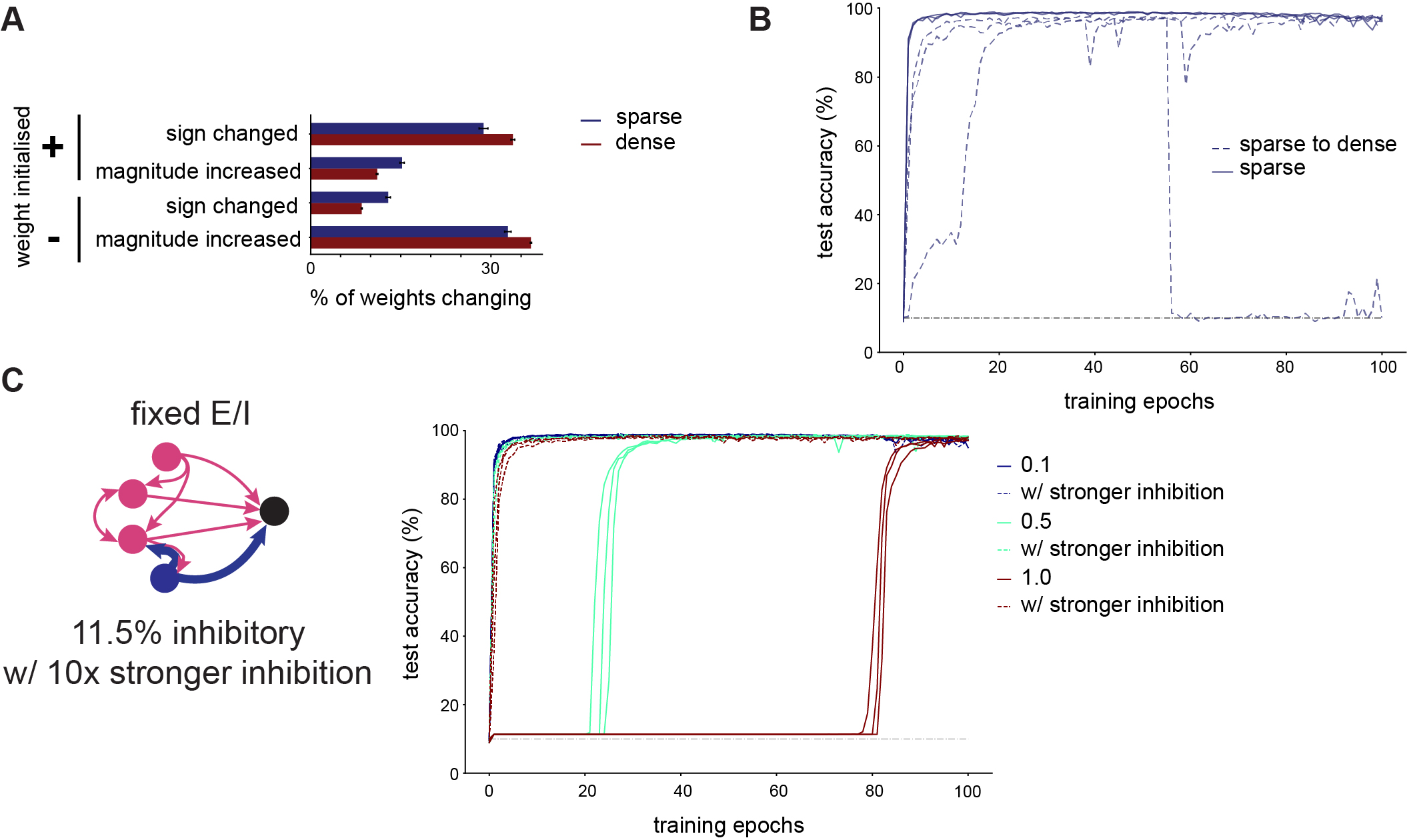


**Fig. S5. Supplement for Dale’s principle figure. A.** Changes in weights after one epoch of training a network with 10000 hidden layer nodes with dense (connection probability = 1, red) or sparse (connection probability = 0.1, blue) connectivity on the MNIST dataset. We illustrate weights whose sign changed (from positive to negative or vice versa), and those whose magnitude increased without changing sign. **B.** Test accuracy over time in large networks (10000 hidden layer nodes) trained on MNIST with fixed excitation and inhibition (11.5% inhibitory nodes). We compare networks which were initialised with a connection probability of 0.1. In sparse networks, any weight that was initialised at zero according to the connection probability was not trainable, and could not change during training. In sparse to dense networks, although the network was initialised with a sparse connectivity matrix, all weights were trainable, and could therefore change from zero, making the network less sparse during training. **C.** We constructed recurrent ANNs where each node was fixed to either excitatory (only positive outgoing weights) or inhibitory (only negative outgoing weights). We set 11.5% of nodes to be inhibitory, as reported in measurements from somatosensory cortex. Inhibitory node outgoing weights were initialised at 10 times the magnitude of excitatory outgoing weights to attempt to counteract the E-I imbalance resulting from the unequal proportion of excitatory and inhibitory nodes. Test accuracy over time in large networks (10000 hidden layer nodes) trained on MNIST with different hidden layer connection probabilities, analogous to Fig. 6B.

## Supplementary Table Headings

**Table S1.** Mean test accuracy after one training epoch on the MNIST dataset for recurrent ANNs with different numbers of hidden layer nodes (rows) and different connection probabilities between nodes in the hidden layer (columns).

**Table S2.** Mean test accuracy after one training epoch on the CIFAR10 dataset for recurrent ANNs with different numbers of hidden layer nodes (rows) and different connection probabilities between nodes in the hidden layer (columns).

**Table S3.** Mean test accuracy after one training epoch on the Sleep-EDF dataset for recurrent ANNs with different numbers of hidden layer nodes (rows) and different connection probabilities between nodes in the hidden layer (columns).
